# Validation of the SACOV-19 score for identifying patients at risk of complicated or more severe COVID-19: a prospective study

**DOI:** 10.1007/s15010-023-02041-8

**Published:** 2023-05-11

**Authors:** Ujjwal Mukund Mahajan, Johanna Erber, Parichehr Shamsrizi, Florian Voit, Jakob Vielhauer, Anna-Lena Johlke, Christopher Benesch, Najib Ben Khaled, Felix Reinecke, Wolf-Stephan Rudi, Matthias Klein, Carolin Jakob, Marcus Oswald, Rainer König, Christian Schulz, Julia Mayerle, Hans Christian Stubbe

**Affiliations:** 1 Department of Medicine II, Medizinische Klinik und Poliklinik II, Hospital of the LMU Munich, LMU Klinikum, Campus Großhadern, Marchioninistr. 15, 81377 Munich, Germany; 2 German Center for Infection Research, Partner Site Munich, Munich, Germany; 3 Department of Internal Medicine II, Technical University of Munich, School of Medicine, University Hospital Rechts Der Isar, Munich, Germany; 4 Department for Clinical Immunology of Infectious Diseases, Bernhard Nocht Institute for Tropical Medicine, Hamburg, Germany; 5 German Center for Infection Research, Partner Site Hamburg-Lübeck-Borstel-Riems, Hamburg, Germany; 6 Department of Anesthesiology, Hospital of the LMU Munich, Munich, Germany; 7 Department of Medicine I, Hospital of the LMU Munich, Munich, Germany; 8 Department of Neurology, Hospital of the LMU Munich, Munich, Germany; 9 Department I of Internal Medicine, University Hospital of Cologne, University of Cologne, Cologne, Germany; 10 German Center for Infection Research, Partner Site Bonn-Cologne, Cologne, Germany; 11 Institute for Infectious Diseases and Infection Control, RG Systemsbiology, Jena University Hospital, Jena, Germany

**Keywords:** SACOV-19, COVID-19, SARS-CoV-2, Decision support, Artificial intelligence, Prospective clinical study

## Abstract

**Purpose:**

Identification of patients at risk of complicated or more severe COVID-19 is of pivotal importance, since these patients might require monitoring, antiviral treatment, and hospitalization. In this study, we prospectively evaluated the SACOV-19 score for its ability to predict complicated or more severe COVID-19.

**Methods:**

In this prospective multicenter study, we included 124 adult patients with acute COVID-19 in three German hospitals, who were diagnosed in an early, uncomplicated stage of COVID-19 within 72 h of inclusion. We determined the SACOV-19 score at baseline and performed a follow-up at 30 days.

**Results:**

The SACOV-19 score’s AUC was 0.816. At a cutoff of > 3, it predicted deterioration to complicated or more severe COVID-19 with a sensitivity of 94% and a specificity of 55%. It performed significantly better in predicting complicated COVID-19 than the random tree-based SACOV-19 predictive model, the CURB-65, 4C mortality, or qCSI scores.

**Conclusion:**

The SACOV-19 score is a feasible tool to aid decision making in acute COVID-19.

**Supplementary Information:**

The online version contains supplementary material available at 10.1007/s15010-023-02041-8.

## Introduction

In December 2019, a cluster of severe pneumonia occurred in the city of Wuhan, China. It was caused by a new betacoronavirus, which was later named the *SARS-Coronaviurs-2 (SARS-CoV-2)* and the infectious disease caused by the new virus was termed *coronavirus disease 2019 (COVID-19)* [1, 2]. COVID-19 poses a severe strain on our societies and healthcare systems. As of March 2023, more than 6.8 million deaths related to COVID-19 have been reported since the beginning of the SARS-CoV-2 pandemic in December 2019 and a significant rate of cases continues to require intensive care treatment [3, 4]. Due to the advent of SARS-CoV-2 vaccines, new antiviral drugs, improved evidence-based treatment algorithms, and changing viral biology, overall mortality and morbidity have substantially declined over the past 2 years. Nonetheless, high numbers of COVID-19 cases continue to pose significant health hazards and challenge healthcare systems worldwide: More than 3.7 million cases have been newly diagnosed and in the 4 weeks leading up to 22nd of March 2023, more than 26,000 patients have died worldwide [4, 5].

Course and outcome of COVID-19 are heterogeneous. Most COVID-19 patients exhibit a mild course and can be managed in an outpatient setting. Progress to more severe stages and critical illness often occurs within hours of hospital admission prompting transfer to the intensive care unit (ICU) [6, 7]. Patients presenting with mild COVID-19 or asymptomatic infection who are at risk for clinical deterioration benefit from close monitoring, swift medication, and supportive measures [8]. Therefore, identifying patients at risk in the early stage of the disease is of paramount importance in medical decision-making regarding follow-up, hospitalization, and guidance for medical treatment [9].

Earlier studies evaluated general disease severity scores such as the CURB-65, NEWS2, or qSOFA in COVID-19. Mostly, these scores were validated for risk of progression to severe COVID-19 or death, to guide Intermediate Care or ICU admission in hospitalized patients [10–13].

Scores specifically developed for risk of progression in COVID-19 like the 4C mortality score (4C), COVID-GRAM or Brescia-COVID Respiratory Severity Scale (BCRSS) almost entirely focus on progression to severe respiratory impairment and death not taking the risk for progression into a complicated stage into consideration [14–17]. Exceptions are the CALL score and the Quick COVID-19 Severity Index (qCSI). Both scores were designed to predict the risk for progression to complicated or more severe COVID-19. The CALL score was based on a relatively small patient cohort [18]. More importantly, its performance in predicting the progression to complicated or more severe COVID-19 was poor (AUC 0.622) [19]. The qCSI score was based on a large dataset boosting a high AUC of 0.81 [20].

To facilitate the identification of patients at risk, we designed the Score for the prediction of an Advanced stage of COVID-19 (SACOV-19) score and predictor model in a previous study [21]. The SACOV-19 score and model predict complicated or more severe COVID-19 in patients with acute COVID-19. They are based on a large retrospective dataset from the Lean European Open Survey on SARS-CoV-2 Infected Patients (LEOSS) cohort [22]. In clinical practice, a SACOV-19 prediction of low risk could support outpatient management. A high predicted risk could promote close follow-up, hospitalization or enter risk–benefit assessments regarding medical treatment. The score and model are based on standard parameters, which can be acquired in most outpatient or hospital settings. In the retrospective dataset of the LEOSS cohort, both tools showed excellent performance [21].

With this study, we aimed to validate the SACOV-19 predictor model and clinical score in a prospective multicenter study. In addition, we compared their performance to three established risk assessment tools: besides the SACOV-19 score and predictor model, we included the 4C mortality score (4C), the qCSI, and the CURB-65 score in this analysis. We chose to compare the 4C, because of its good performance and thorough validation, even though it was designed to predict mortality in patients hospitalized with COVID-19. The qCSI score, on the other hand, aimed at predicting the risk of severe adverse events in patients with COVID-19 in the emergency department setting to predict the adverse outcome within 24 h [14, 20, 23]. A qCSI cutoff of higher than three was determined to classify patients with acute COVID-19 at high risk of deterioration [15]. Finally, we selected the CURB-65, which is an established tool for determining the severity and prognosis of patients with pneumonia, but was conceived long before the advent of COVID-19. A CURB-65 of higher than one usually warrants hospital admission [23].

## Methods

### Study design and participants

We designed a prospective multicenter cohort study to validate the SACOV-19 score and the SACOV-19 predictor model [21]. We included patients of 18 years or older, who presented with mild or asymptomatic SARS-CoV-2 infection within 72 h of the first positive SARS-CoV-2 test to a study site during the period 1/2021 to 4/2022. Uncomplicated COVID-19 was defined as asymptomatic SARS-CoV-2 infection, or COVID-19 with upper respiratory tract-associated symptoms, gastrointestinal symptoms, fever, headache, nausea, dizziness, or symptoms of the musculoskeletal system such as joint or muscle pain and absence of the complicated or more severe phase as per the LEOSS definition, corresponding to a WHO Clinical Progression Scale of less than three [22, 24]. We excluded patients presenting in complicated or more severe COVID-19 phases or in recovery at baseline.

The primary endpoints were (1) the composite endpoint of occurrence of complicated or critical COVID-19 as per the LOESS definition or death and (2) reaching the recovery phase without progressing to more severe stages [22, 25]. Patients received a baseline visit at inclusion encompassing a clinical laboratory assessment. The follow-up evaluation was conducted 30 days after the baseline to assess the overall outcome and endpoints.

All study participants were recruited when presenting as in- or outpatients to the clinics or wards of the participating study centers after informed consent was obtained. Three German study centers participated in this study: the LMU Klinikum of the Ludwig Maximilian University of Munich (LMU), the University Hospital Rechts der Isar in Munich (MRI), and the University Medical Center Hamburg-Eppendorf (UKE) in Hamburg. This study was approved by the ethics committees of the Medical Faculties of the LMU Munich, the Technical University Munich, and the University of Hamburg. The study was registered at the German Clinical Trials Register (DRKS) DRKS-ID DRKS00023896.

### Data collection

At baseline, the attending study physician determined the disease severity following the LEOSS criteria. The SACOV-19 items, demographic and clinical data as well as laboratory parameters were collected using the electronic data capture (EDC) software LCARS-C (LMU Klinikum, Germany) at baseline. The follow-ups were conducted 30 days after baseline. If the study participants were discharged or not admitted at all, the study personnel (i.e., the study nurse or study physician) contacted the patient 30 days after baseline to determine the outcome.

### Scores and models

To assess the SACOV-19 score and model, the 16 parameters of the predictor model (11 of which are required to calculate the SACOV-19 score) were recorded (see supplementary table 1 for all score items). In addition, parameters for established risk scores and new COVID-19-associated scores, such as CURB-65, qCSI, and 4C were recorded at baseline. The results of each score and the SACOV-19 model were computed using R 4.2.2 after the completion of the study [26]. The study personnel was blinded to the scoring results to reduce bias and to avoid incorporating non-validated scoring results into routine practice.

### Statistical analysis

Numeric variables are represented as medians with interquartile ranges (IQR). To test for statistically significant differences of medians between groups, we used a two-sided Kruskal–Wallis test. Categorical variables are displayed as counts with percentages. To test for statistically significant differences in count data, we used Pearson's Chi-squared test. Diagnostic test performance was assessed by calculating sensitivity, specificity, positive predictive values (PPV), negative predictive values (NPV), accuracies, and receiver operating characteristic (ROC) curves with their respective area under the curve (AUC). The respective results are expressed together with their 95% confidence intervals (CI). All statistical analyses and data visualization were carried out with R 4.2.2 [26].

## Results

### Study participants

**Fig. 1:**
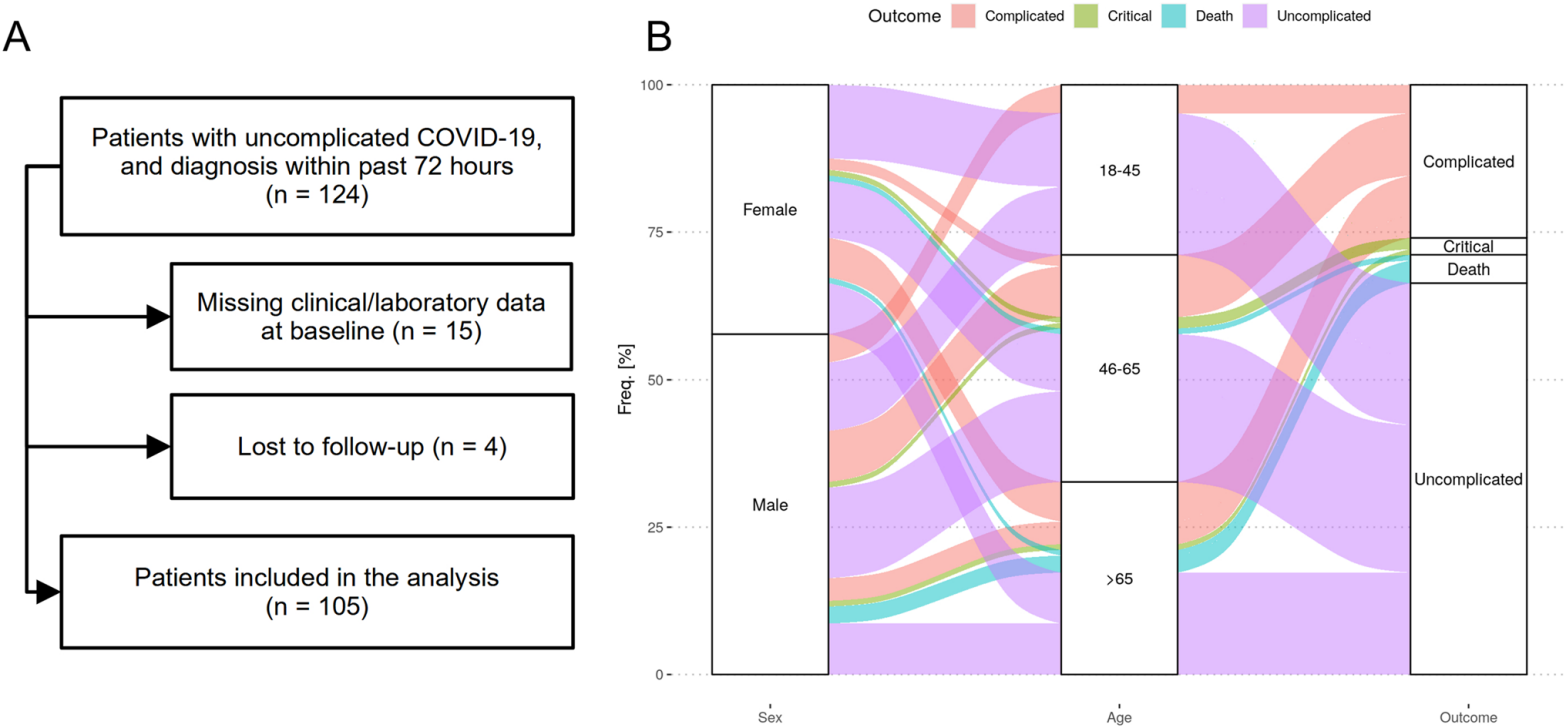
Study flow chart and patient characteristics. **A** The diagram indicates the study’s patient inclusion and selection. **B** The chart depicts the distribution of sex, age, and outcome in the study cohort in an alluvial diagram

We included 124 patients with acute COVID-19, who were PCR-confirmed within the 72 h prior to the baseline visit in three university hospitals (LMU *n* = 86, MRI *n* = 30, and UKE *n* = 8). In 15 participants, essential baseline variables for computing the SACOV-19 score and model were missing. These patients were excluded from the analysis. Four participants were lost to follow-up and had to be removed from the analysis. In total, the analysis could be completed in 105 participants (Fig. 1A).

### Clinical characteristics

We compared the characteristics of patients progressing to complicated or more severe disease (in the following referred to as *progressors*) with patients who did not experience complicated or more severe COVID-19 during the 30-day follow-up (in the following referred to as *non-progressors*). Progressors were significantly older than non-progressors with a median age of 65.0 [52.0; 77.5] years compared to 52.5 [38.2; 65.8] years (*p* = 0.007). They tended towards a higher body mass index (BMI) of 28.7 [24.7; 31.2] kg/cm^2^ compared to 25.4 [22.4; 29.3] kg/cm^2^ (*p* = 0.064). No significant differences were observed for sex, smoker status, preexisting health conditions, or SARS-CoV-2 vaccine status. While most vital parameters did not show significant differences, progressors exhibited a slight, but consistent lower peripheral oxygen saturation at baseline of 94% [93%; 95%] compared to 96% [95%; 98%] (*p* < 0.001). They tended to present with a higher respiratory rate at baseline with 18 [16; 19] min^−1^ compared to 16 [14; 18] min^−1^ (*p* = 0.067). Progressors reported the symptom dyspnea more frequently with 47.1% compared to 22.4% (*p* = 0.021). No significant differences were observed for other symptoms. In the laboratory data, progressors exhibited higher inflammatory markers such as CRP with 5.30 [3.35; 9.15] mg/dl compared to 2.00 [0.60; 4.53] mg/dl (*p* < 0.001) and IL6 of 56.0 [30.9; 89.3] pg/ml compared to 15.4 [9.25; 45.2] pg/ml (*p* < 0.001). Additionally, significantly elevated values were observed in progressors compared to non-progressors for creatinine (1.10 [0.90; 1.75] mg/dl compared to 0.90 [0.70; 1.28] mg/dl; *p* = 0.01), LDH (313 [256; 428] U/l compared to 246 [213; 306] U/l; *p* = 0.002) and troponin T (18.5 [13.0; 36.5] ng/ml compared to 13.0 [13.0; 14.0] ng/ml; *p* = 0.015). The patient characteristics are summarized in Table 1.

**Table 1:** Summary of patient characteristics

Variable	Non-progressor	Progressor	*p* value
	*N* = 70	*N *= 35	
Age [years]	52.5 [38.2;65.8]	65.0 [52.0;77.5]	0.007
Sex at birth			0.332
Female	32 (46.4%)	12 (34.3%)	
Male	37 (53.6%)	23 (65.7%)	
BMI [kg/m^2^]	25.4 [22.4;29.3]	28.7 [24.7;31.2]	0.064
Smoker status			0.519
Current	6 (16.2%)	2 (13.3%)	
Former	7 (18.9%)	5 (33.3%)	
Never	24 (64.9%)	8 (53.3%)	
Solid tumors			1.0
Localized	6 (8.82%)	3 (8.57%)	
Metastatic	1 (1.47%)	0 (0.00%)	
Hematologic malignancy	1 (1.47%)	3 (8.57%)	0.113
Arterial hypertension	20 (31.2%)	12 (34.3%)	0.933
Rheumatic disease	2 (2.99%)	1 (2.86%)	1.0
Acute kidney injury	4 (6.35%)	3 (9.09%)	0.689
GCS	15.0 [15.0;15.0]	15.0 [15.0;15.0]	0.461
Blood pressure, systolic [mmHg]	130 [120;142]	133 [121;146]	0.869
Pulse frequency 1/min	84.5 [75.0;95.0]	81.0 [76.0;87.8]	0.488
Temperature [°C]	37.3 [36.7;38.0]	37.9 [37.0;38.8]	0.146
SpO2 [%]	96.0 [95.0;98.0]	94.0 [93.0;95.0]	< 0.001
Respiratory rate [1/min]	16.0 [14.0;18.0]	18.0 [16.0;19.0]	0.067
Dyspnea	15 (22.4%)	16 (47.1%)	0.021
Headache	11 (15.9%)	7 (20.6%)	0.758
Diarrhea	3 (4.41%)	5 (15.2%)	0.109
Hemoptysis	1 (1.49%)	1 (3.12%)	0.544
Unconsciousness	2 (3.03%)	0 (0.00%)	0.551
Cough	29 (42.6%)	17 (50.0%)	0.622
Nausea	10 (14.9%)	8 (25.0%)	0.349
ALT [U/l]	30.0 [20.0;46.5]	25.0 [19.0;40.5]	0.793
AST [U/l]	34.0 [24.8;55.5]	35.0 [29.5;49.5]	0.407
Bilirubin (direct) [mg/dl]	0.30 [0.20;0.45]	0.40 [0.27;0.58]	0.498
BUN [mg/dl]	13.5 [7.78;18.5]	24.0 [16.0;32.0]	0.024
Creatinine [mg/dl]	0.90 [0.70;1.28]	1.10 [0.90;1.75]	0.010
CRP [mg/dl]	2.00 [0.60;4.53]	5.30 [3.35;9.15]	< 0.001
D-dimer [mg/dl]	0.90 [0.50;2.89]	0.90 [0.60;2.55]	0.683
Gamma-GT [U/l]	36.0 [22.5;52.0]	61.0 [28.0;106]	0.025
Interleukin-6 [pg/ml]	15.4 [9.25;45.2]	56.0 [30.9;89.3]	< 0.001
INR	1.00 [0.90;1.00]	0.90 [0.90;1.00]	0.746
LDH [U/l]	246 [213;306]	313 [256;428]	0.002
Lymphocyte count [G/l]	1.09 [0.83;1.45]	0.86 [0.58;1.41]	0.197
Neutrophils count [G/l]	3.12 [2.50;4.65]	3.14 [2.70;5.09]	0.656
Troponin T [ng/ml]	13.0 [13.0;14.0]	18.5 [13.0;36.5]	0.015
Completed SARS-CoV-2 vaccination	15 (21.7%)	10 (28.6%)	0.571

### Outcomes

The outcome of the study participants was assessed 30 days after baseline. During the follow-up period, 35 (33%) patients progressed to a complicated or more severe COVID-19 stage: 27 patients progressed to the complicated and three to a critical stage, while five patients died. (Fig. 1B). In progressors, the primary composite endpoint of complicated or more severe COVID-19 occurred at a median of 5 [1; 12] days after baseline.

### SACOV-19 performance

To assess the performance of the SACOV-19 score and predictor model, we computed score results and model predictions to the outcome at 30 days.

**Fig. 2:**
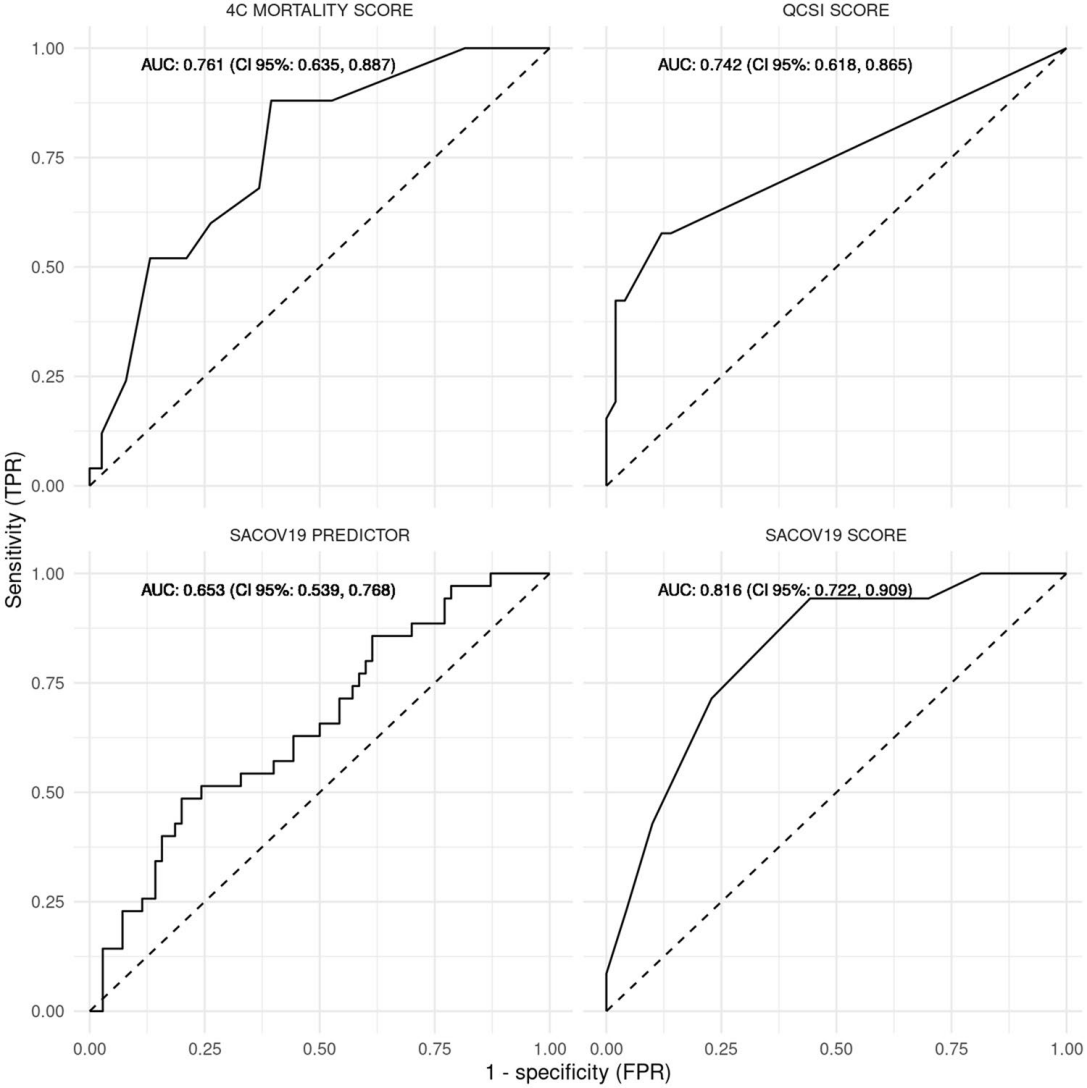
ROC curves and AUCs. ROC curves were plotted for the SACOV-19 score and predictor as well as the 4C mortality score and the qCSI. The dashed lines represent the chance lines, where the AUC (area under the curve) would be 0.5, corresponding to a balanced random prediction. AUCs are given with 95% CI

**Fig. 3:**
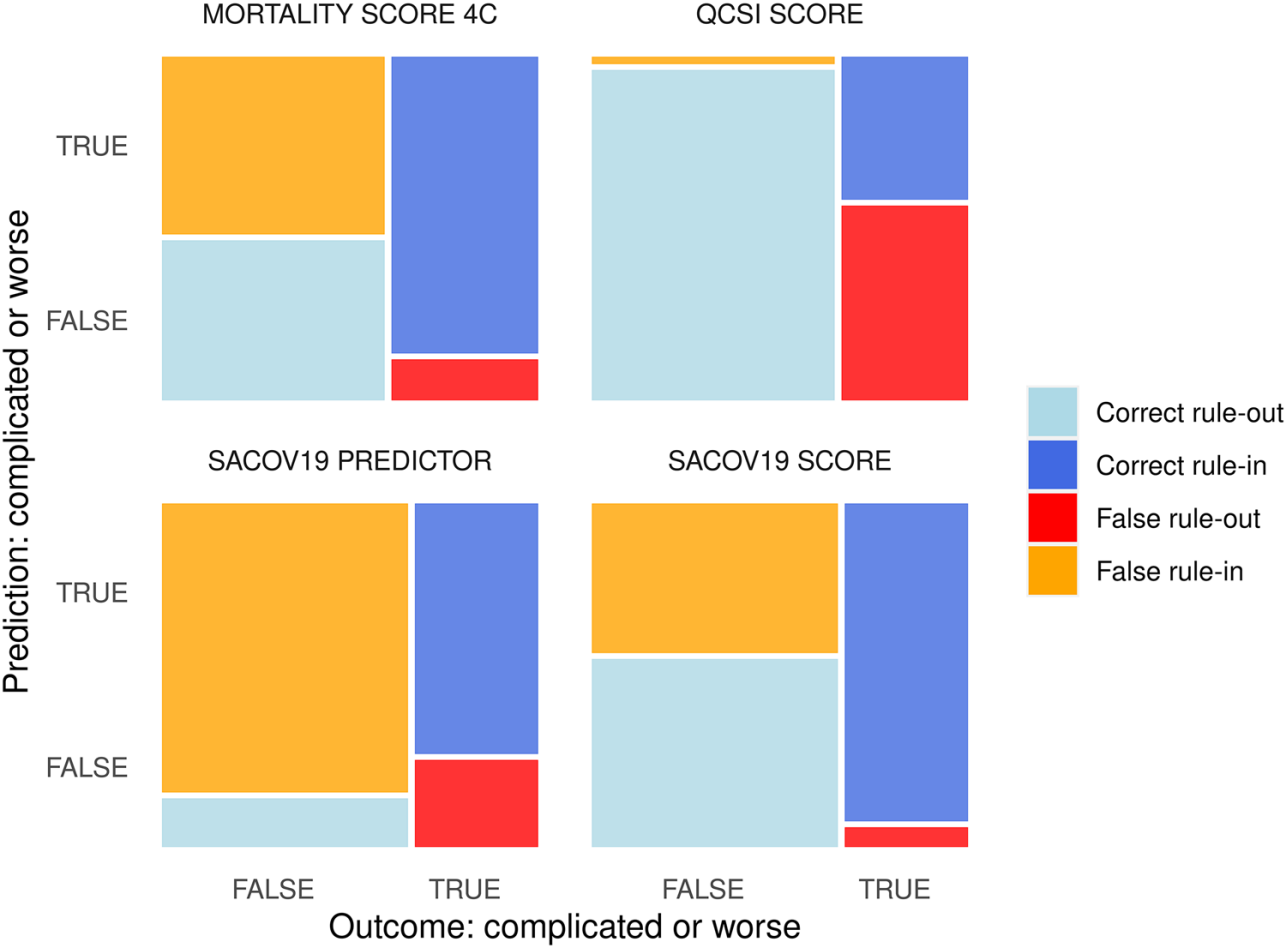
Comparison of SACOV-19 score and predictor. Mosaic diagram with each field’s size corresponding to the count of correct and false rule-in and -out (i.e., in analogy to the four fields of the respective contingency tables). False rule-out in red signifies missed adverse outcomes and the correct rule-out in dark blue corresponds to correctly identified cases with complicated or worse outcomes

The performance of the SACOV-19 score was better than the predictor model with an AUC of 0.816 (CI 95% 0.722, 0.909) compared to 0.653 (CI 95% 0.539, 0.768; Fig. 2). At the suggested cutoff of > 3, the SACOV-19 score exhibited an accuracy of 0.67 (CI 95% 0.59, 0.77) with a sensitivity of 0.943 (CI 95% 0.888, 0.997), a specificity of 0.557 (CI 95% 0.393, 0.722), an NPV of 0.952 (CI 95% 0.899, 1.000) and a PPV of 0.512 (CI 95% 0.395, 0.665). It performed significantly better than the SACOV-19 predictor model, which had an accuracy of 0.343 (CI 95% 0.253, 0.442), a sensitivity of 0.743 (CI 95% 0.640; 0.845), a specificity of 0.143 (CI 95% 0.027, 0.259), an NPV of 0.530 (CI 95% 0.425, 0.636) and a PPV of 0.299 (CI 95% 0.093, 0.505; Table 2, Fig. 3). In our cohort, the SACOV-19 score correctly predicted an uncomplicated outcome in 39 of 70 non-progressors (56%), while wrongly predicting an uncomplicated course in only two of 35 progressors (5.7%). The predictor model would have predicted an uncomplicated outcome in 10 of 70 non-progressors (14%). Of the 35 progressors, nine (26%) received the wrong prediction (uncomplicated outcome).

**Table 2:** Score performances

Parameter	SACOV-19 score	SACOV-19 predictor	qCSI	4C	CURB-65
AUC	0.816 (0.722, 0.909)	0.653 (0.539, 0.768)	0.742 (0.618, 0.865)	0.761 (0.635, 0.887)	0.624 (0.480, 0.769)
Accuracy	0.686 (0.588, 0.773)	0.343 (0.253, 0.442)	0.789 (0.681, 0.875)	0.635 (0.504, 0.753)	0.631 (0.502, 0.747)
Sensitivity	0.943 (0.888, 0.997)	0.743 (0.640, 0.845)	0.423 (0.286, 0.560)	0.880 (0.777, 0.983)	0.000 (0.000, 0.000)
Specificity	0.557 (0.393, 0.722)	0.143 (0.027, 0.259)	0.980 (0.926, 1.000)	0.474 (0.278, 0.669)	1.000 (1.000, 1.000)
PPV	0.512 (0.359, 0.665)	0.299 (0.093, 0.505)	0.912 (0.843, 0.982)	0.452 (0.239, 0.664)	NA
NPV	0.952 (0.899, 1.000)	0.530 (0.425, 0.636)	0.775 (0.539, 1.000)	0.889 (0.794, 0.984)	NA

For each score, AUC, accuracy, sensitivity, specificity, PPV, and NPV were calculated. Results are given with the CI 95%

This table shows performance measures (95% confidence interval) for the included scores. AUC (area under the curve), SACOV-19 (Score for the prediction of an Advanced stage of COVID-19), qCSI (Quick COVID-19 Severity Index), 4C (4C mortality score), PPV (positive predictive value), NPV (negative predictive value)

Finally, we evaluated the performances of the 4C mortality, the qCSI and the CURB-65 scores. The 4C and the qCSI showed AUCs of 0.761 (CI 95% 0.635, 0.887) and 0.742 (CI 95% 0.618, 0.865), respectively. At a cutoff of greater than three for both, their accuracies were 0.635 (CI 95% 0.504, 0.7527) and 0.789 (CI 95% 0.681, 0.875). The 4C had a sensitivity of 0.880 (CI 95% 0.777, 0.983), with a specificity of 0.474 (CI 95% 0.278, 0.669). The qCSI score had a sensitivity of 0.423 (CI 95% 0.286, 0.560) while exhibiting a high specificity with 0.980 (CI 95% 0.926, 1.000; Fig. 3). The PPVs for the scores were 0.452 (CI 95% 0.239, 0.664) and 0.912 (0.843, 0.982), respectively. The corresponding NPVs were 0.889 (CI 95% 0.794, 0.984) and 0.775 (CI 95% 0.539, 1.000).

At baseline, the CURB-65 awarded all patients in our cohort a score of less than two (i.e., eligible for outpatient treatment). Consequently, its sensitivity was zero and its specificity one for predicting adverse outcomes not adding any diagnostic value in this study’s setting (Table 2).

## Discussion

The SACOV-19 score and the predictive model were designed to guide the identification of patients at risk of adverse outcomes in COVID-19 [21]. To enable their use in clinical practice as decision support, a prospective validation was required. To this end, we conducted a prospective multicenter validation study. We analyzed 105 patients who were diagnosed with uncomplicated COVID-19 within 72 h of baseline. Altogether, 35 of them developed an adverse outcome (i.e., occurrence of complicated or more severe COVID-19) during a 30-day follow-up. The SACOV-19 score performed significantly better than the SACOV-19 predictive model with an AUC of 0.816 (CI 95% 0.722, 0.909). At the cutoff of > 3, the score was suited for ruling-out clinical deterioration with a high sensitivity (94%) and an acceptable specificity (56%).

We compared the SACOV-19 score’s ability to predict adverse outcomes to other established predictive scores: 4C, qCSI, and CURB-65. The 4C was designed to predict mortality in patients hospitalized with COVID-19. It was published with an AUC of 0.79 (95% CI 0.78, 0.79) for predicting mortality in hospitalized patients [14]. In a retrospective, external validation, its AUC was 0.77 (CI 95%, 0.79, 0.87) [27]. Here, we tested its ability to predict complicated or more severe COVID-19. In this setting, its AUC was 0.761 (CI 95% 0.635, 0.887), similar to the previously published AUC indicating good discriminatory power [14, 27]. The qCSI was designed to predict any adverse outcome in patients presenting to the emergency department with COVID-19 within 24 h [20]. In our study, its AUC was 0.742 (CI 95% 0.618, 0.865) for adverse events at 30 days after baseline. This was lower compared to its published AUC of 0.81 (CI 95% 0.73, 0.89) and the SACOV-19 scores [20]. While the AUC of the qCSI was in a good range, its other performance markers at the suggested cutoff of greater than three were not suited for ruling out progression to more complicated COVID-19, but rather optimized on specificity and a high PPV. The CURB-65 is an established score for risk stratification in patients with pneumonia [23]. In the setting of this study, the CURB-65 was < 2 in all patients classifying all as fit for discharge. Therefore, the CURB-65 was removed from the analysis. Interestingly, the 4C, designed to predict mortality, performed similarly to the qCSI score, while the CURB-65 performed worst. The latter was not optimized for COVID-19. The qCSI only incorporated clinical features but no laboratory values in an entity with ever-changing clinical presentation [28]. In contrast, the SACOV-19 score and the 4C additionally incorporate laboratory parameters, which seem to boost their predictive power in this setting.

Our study has important limitations: We conducted an a priori power analysis for the primary end-point (i.e., the composite endpoint of occurrence of complicated or critical COVID-19 as per the LOESS definition or death; see supplementary information). Therefore, this study was only powered to validate the SACOV-19 score and predictive model for the occurrence of complicated or more severe COVID-19, but not mortality. Given the occurrence of only five deaths during the 30-day follow-up, valid estimates of the score’s ability to predict mortality in COVID-19 are not possible. Another limitation is that the viral variants were not included in the analysis. However, the timeframe of the study indicates, that several variants including the omicron lineage BA.2 circulating in 4/2022 were included suggesting a robust predictive power across SARS-CoV-2 variants [29].

Notably, SACOV-19 was developed based on data from patients with older variants (between 3/2020 and 7/2020). Hence, this study underlines its applicability to newer strains. Since the SACOV-19 score was established taking only patients from the LEOSS dataset into account, a very strict inclusion was applied allowing only participants to enter the study who were diagnosed with PCR-confirmed SARS-CoV-2 infection within 72 h prior to the baseline visit. This was to avoid the bias of including patients in more advanced stages of the infection. The scores performance in more advanced, but uncomplicated stages remains unclear. Finally, the SACOV-19 score has been established and is now validated in German cohorts. While our findings support its use in German and similar settings as well as across a variety of existing variants, further validation should be carried out as new variants, treatments, and prevention strategies emerge, ideally in an international setting.

## Conclusion

This study externally and prospectively validates the SACOV-19 score as an accurate tool to identify patients at risk of clinical deterioration who are diagnosed with asymptomatic SARS-CoV-2 or mild COVID-19. Most predictive tools for the outcome of COVID-19 focus on hospitalized patients and the prediction of mortality [17]. Therefore, external validation of the SACOV-19 score is an important step towards reliable and robust decision-making tools. It supports the score’s use in clinical practice when deciding for the degree of monitoring of patients. In the future, SACOV-19 might also guide risk–benefit evaluations of treatment strategies.

## Supplementary Information

Below is the link to the electronic supplementary material.

Supplementary file1 (DOCX 44 KB)

## Data Availability

The clinical dataset will be made available upon reasonable request to the corresponding authors.
