# Supplementary material for: Validation of the SACOV-19 score for identifying patients at risk of complicated or more severe COVID-19: a prospective study
Source: Infection. 2023 May 11;51(6):1669–78. doi: 10.1007/s15010-023-02041-8 (PMC10173210; doi:10.1007/s15010-023-02041-8)
Supplement: Supplementary file 1 — Supplementary file1 (DOCX 44 KB) [file 15010_2023_2041_MOESM1_ESM.docx]

# Validation of the SACOV-19 score for identifying patients at risk of complicated or more severe COVID-19: a prospective study

**Table of content**

Supplementary tables 1

Sample size calculation 2

References 3

## Supplementary tables

Supplementary table 1. Variables for baseline assessment of scores

| **Variable** | **Type** | **SACOV-19 predictor** | **SACOV-19 score** | **CURB-65** | **qCSI** |
| --- | --- | --- | --- | --- | --- |
| Age | general | x | x | x |  |
| Sex | general |  |  |  |  |
| BMI | general | x | x |  |  |
| Smoking status | general | x | x |  |  |
| CRP | lab | x | x |  |  |
| LDH | lab | x | x |  |  |
| Lymphocyte count | lab | x | x |  |  |
| Neutrophils count | lab | x |  |  |  |
| Creatinine | lab | x |  |  |  |
| AST | lab | x |  |  |  |
| Gamma-GT | lab | x |  |  |  |
| BUN | lab |  |  | x |  |
| pO2 | lab |  |  |  |  |
| ALT | lab | x |  |  |  |
| Dyspnea | symptoms | x | x |  |  |
| Confusion | symptoms |  |  | x |  |
| Oxygen supplementation | treatment |  |  |  | x |
| Respiratory rate | vitals | x | x | x | x |
| Oxygen saturation | vitals | x | x |  | x |
| Temperature | vitals | x | x |  |  |
| Systolic blood pressure | vitals |  |  | x |  |
| Heart rate | vitals |  |  |  |  |
| Acute kidney injury | complications | x | x |  |  |

Variables required for different scoring systems.

**Supplementary table 2. Variables for the assessment of COVID-19 phases**

| **Variable** | **complicated** | **critical** | **recovery** |
| --- | --- | --- | --- |
| need for new oxygen supplementation | x |  |  |
| clinically meaningful increase of prior oxygen home therapy | x |  |  |
| PaO2 at room air < 70 mmHg | x |  |  |
| SO2 at room air < 90 % | x |  |  |
| AST or ALT > 5x ULN | x |  |  |
| new cardiac arrhythmia | x |  |  |
| new pericardial effusion > 1 cm | x |  |  |
| new heart failure with pulmonary edema, congestive hepatopathy or peripheral edema | x |  |  |
| need for catecholamines |  | x |  |
| life-threatening cardiac arrhythmia |  | x |  |
| need for unplanned mechanical ventilation (invasive or non-invasive) |  | x |  |
| prolongation (>24h) of planned mechanical ventilation |  | x |  |
| Liver failure with Quick < 50 % or INR > 3.5 |  | x |  |
| qSOFA >= 2 |  | x |  |
| acute renal failure in need of dialysis |  | x |  |
| improvement by one degree of severity according to this scheme or discharge from hospital |  |  | x |
| defervescence |  |  | x |
| no further progression or re-hospitalization |  |  | x |

Variables for assessing the COVID-19 phase at baseline and follow-up according to LEOSS [1].

## Sample size calculation

To determine the required sample size we conducted an a priori sample size calculation for the primary endpoint “occurrence of complicated or more severe COVID-19” before commencing the study. We used the R package pROC implementing the methodology suggested by Obuchowski et al. [2, 3]. Given an AUC of 0.80 of the predictor model [4], a power of 0.8 and a significance level of 0.05, a minimum of 13 complicated or critical COVID-19 cases was required. In the Lean European Open Survey on SARS-CoV-2-Infected Patients (LEOSS) cohort, the overall prevalence of complicated or more severe COVID‑19 was 35% (755/2,155) [1]. In the COVID-19 registry of the LMU Klinikum (CORKUM; data not published), we observed 21% (82/393) of complicated cases. At a rate of 21% to 35%, we estimated that we would require a minimum total number of 62 patients. Accounting for variance in disease severity over time and a potential loss of follow-up, we aimed at including a minimum of 100 patients.

## References

1. Jakob CEM, Borgmann S, Duygu F, Behrends U, Hower M, Merle U, Friedrichs A, Tometten L, Hanses F, Jung N, Rieg S, Wille K, Grüner B, Klinker H, Gersbacher-Runge N, Hellwig K, Eberwein L, Dolff S, Rauschning D, von Bergwelt-Baildon M, Lanznaster J, Strauß R, Trauth J, de With K, Ruethrich M, Lueck C, Nattermann J, Tscharntke L, Pilgram L, Fuhrmann S, Classen A, Stecher M, Schons M, Spinner C, Vehreschild JJ (2020) First results of the “Lean European Open Survey on SARS-CoV-2-Infected Patients (LEOSS).” Infection. https://doi.org/10.1007/s15010-020-01499-0

2. Robin X, Turck N, Hainard A, Tiberti N, Lisacek F, Sanchez J-C, Müller M, code) SS (Fast D, Multiclass) MD (Hand & T (2020) pROC: Display and Analyze ROC Curves

3. Obuchowski NA, Lieber ML, Wians FH (2004) ROC Curves in Clinical Chemistry: Uses, Misuses, and Possible Solutions. Clin Chem 50:1118–1125. https://doi.org/10.1373/clinchem.2004.031823

4. Jakob CEM, Mahajan UM, Oswald M, Stecher M, Schons M, Mayerle J, Rieg S, Pletz M, Merle U, Wille K, Borgmann S, Spinner CD, Dolff S, Scherer C, Pilgram L, Rüthrich M, Hanses F, Hower M, Strauß R, Massberg S, Er AG, Jung N, Vehreschild JJ, Stubbe H, Tometten L, König R (2021) Prediction of COVID-19 deterioration in high-risk patients at diagnosis: an early warning score for advanced COVID-19 developed by machine learning. Infection 1–12. https://doi.org/10.1007/s15010-021-01656-z
